# Supplementary material for: ProteinShader: illustrative rendering of macromolecules
Source: BMC Struct Biol. 2009 Mar 30;9:19. doi: 10.1186/1472-6807-9-19 (PMC2672931; doi:10.1186/1472-6807-9-19)
Supplement: Additional file 1 — ProteinShader program without source code. This compressed file contains the complete ProteinShader program including associated libraries, but no source code. A README.txt file gives an overview of the ProteinShader distribution, and the index.html file in the help subdirectory has directions on getting started with the program as well as a set of tutorials. [file 1472-6807-9-19-S1.zip › ProteinShader-beta-0_9_4-binary/help/api/org/proteinshader/gui/viewing/Camera.html]

Camera (ProteinShader API)


|  |  |  |
| --- | --- | --- |
| **Overview** \| **Package** \| **Class** \| **Use** \| **Tree** \| **Deprecated** \| **Index** \| **Help** | |  |
| PREV CLASS   **NEXT CLASS** | **FRAMES**    **NO FRAMES**     **All Classes** |  |
| SUMMARY: NESTED \| FIELD \| CONSTR \| METHOD | DETAIL: FIELD \| CONSTR \| METHOD |  |


---


## org.proteinshader.gui.viewing Class Camera

```
java.lang.Object
  org.proteinshader.gui.viewing.Camera
```

---

``` public class Camera extends Object ```

Used to store and manipulate camera information.

---

| **Field Summary** | |
| --- | --- |
| `static double` | `FOVY`             The fovy (field of view on y-axis) for the camera is 60 degrees. |
| `static double` | `HALF_FOVY`             Half the fovy is useful for some calculations. |
| `static double` | `HALF_FOVY_RADIANS`             Half the fovy needs to be in radians for Java trig functions. |
| `static int` | `MIN_INITIAL_CAMERA_DISTANCE`             The minimum initial camera distance is 20. |
| `static double` | `SIN_HALF_FOVY`             The sine of HALF\_FOVY\_RADIANS is used in pixel-angstrom conversions. |
| `static double` | `TAN_HALF_FOVY`             The tangent of HALF\_FOVY\_RADIANS is used in pixel-angstrom conversions. |


**Constructor Summary**

`Camera()`
  
          Constructs a Renderer.


| **Method Summary** | |
| --- | --- |
| `void` | `aim(GL gl)`             Uses gluLookAt() to position and aim the camera. |
| `double` | `calculateDepth(double x, double y, double z)`             Calculates the depth (z-coordinate in camera space) of the xyz-center of the Drawable object. |
| `double` | `calculateDepth(Drawable d)`             Calculates the depth (z-coordinate in camera space) of the xyz-center of the Drawable object. |
| `double` | `calculateDistance(double x, double y, double z)`             Calculates the distance between the camera and the xyz-center of the Drawable object by using the Pythagorean theorem. |
| `double` | `calculateDistance(Drawable d)`             Calculates the distance between the camera and the xyz-center of the Drawable object by using the Pythagorean theorem. |
| `double` | `convertToAngstroms(double pixels, double cameraDepth)`             Converts pixels to angstroms based on depth in camera space. |
| `void` | `initializeViewport(GLAutoDrawable canvas, GL gl)`             Sets the viewport so that the origin (0, 0, 0) is in its center. |
| `void` | `moveX(int x)`             Add to the camera x position. |
| `void` | `moveY(int y)`             Add to the camera y position. |
| `void` | `moveZ(int z)`             Add to the camera z position. |
| `void` | `printModelViewMatrix()`             This debugging method prints the copy of the ModelViewMatrix stored n m\_modelViewMatrix. |
| `void` | `printXYZ()`             Prints the xyz-coordinates for the camera. |
| `void` | `resetDistance()`             Sets the camera position based on the initial camera distance calculated when a new Structure is loaded. |
| `void` | `reshape(GL gl, int x, int y, int width, int height)`             Called on when the GLAutoDrawable object has changed its size so that the perspective and viewport can be recalculated. |
| `void` | `setAccPerspective(GLAutoDrawable canvas, GL gl, double dxPixels, double dyPixels)`             Sets the projection matrix by using the glFrustrum() method of OpenGL. |
| `void` | `setGluPerspective(GLAutoDrawable canvas, GL gl)`             Sets the projection matrix by using the gluPerspective() method of OpenGL. |
| `void` | `setInitialCameraDistance(int z)`             Sets the initial camera distance on the z-axis. |
| `void` | `updateModelViewMatrix(GL gl)`             Updates a copy of the OpenGL model view matrix. |

**Methods inherited from class java.lang.Object**

`clone, equals, finalize, getClass, hashCode, notify, notifyAll, toString, wait, wait, wait`

**Field Detail**

### FOVY

```
public static final double FOVY
```

:   The fovy (field of view on y-axis) for the camera is 60 degrees.

    **See Also:**: Constant Field Values

---


### HALF\_FOVY

```
public static final double HALF_FOVY
```

:   Half the fovy is useful for some calculations.

    **See Also:**: Constant Field Values

---


### HALF\_FOVY\_RADIANS

```
public static final double HALF_FOVY_RADIANS
```

:   Half the fovy needs to be in radians for Java trig functions.

    **See Also:**: Constant Field Values

---


### SIN\_HALF\_FOVY

```
public static final double SIN_HALF_FOVY
```

:   The sine of HALF\_FOVY\_RADIANS is used in pixel-angstrom conversions.

---


### TAN\_HALF\_FOVY

```
public static final double TAN_HALF_FOVY
```

:   The tangent of HALF\_FOVY\_RADIANS is used in pixel-angstrom
    conversions.

---


### MIN\_INITIAL\_CAMERA\_DISTANCE

```
public static final int MIN_INITIAL_CAMERA_DISTANCE
```

:   The minimum initial camera distance is 20.

    **See Also:**: Constant Field Values


**Constructor Detail**

### Camera

```
public Camera()
```

:   Constructs a Renderer.


**Method Detail**

### updateModelViewMatrix

```
public void updateModelViewMatrix(GL gl)
```

:   Updates a copy of the OpenGL model view matrix. This method should be
    called before using the calculateCameraDistance() method.

    :   **Parameters:**: `gl` - the current GL object.

---


### calculateDistance

```
public double calculateDistance(Drawable d)
```

:   Calculates the distance between the camera and the xyz-center of the
    Drawable object by using the Pythagorean theorem.

---


### calculateDistance

```
public double calculateDistance(double x,
                                double y,
                                double z)
```

:   Calculates the distance between the camera and the xyz-center of the
    Drawable object by using the Pythagorean theorem.

---


### calculateDepth

```
public double calculateDepth(Drawable d)
```

:   Calculates the depth (z-coordinate in camera space) of the xyz-center of
    the Drawable object.

---


### calculateDepth

```
public double calculateDepth(double x,
                             double y,
                             double z)
```

:   Calculates the depth (z-coordinate in camera space) of the xyz-center of
    the Drawable object.

---


### convertToAngstroms

```
public double convertToAngstroms(double pixels,
                                 double cameraDepth)
```

:   Converts pixels to angstroms based on depth in camera space.

    :   **Parameters:**: `pixels` - the number of pixels to convert to angstroms.: `cameraDepth` - the z-value of an object in camera space. **Returns:**: The equivalent number of angstroms.

---


### initializeViewport

```
public void initializeViewport(GLAutoDrawable canvas,
                               GL gl)
```

:   Sets the viewport so that the origin (0, 0, 0) is in its center.

    :   **Parameters:**: `canvas` - the canvas to draw on.: `gl` - the current GL object.

---


### setGluPerspective

```
public void setGluPerspective(GLAutoDrawable canvas,
                              GL gl)
```

:   Sets the projection matrix by using the gluPerspective() method of OpenGL.

    :   **Parameters:**: `canvas` - the canvas to draw on.: `gl` - the current GL object.

---


### setAccPerspective

```
public void setAccPerspective(GLAutoDrawable canvas,
                              GL gl,
                              double dxPixels,
                              double dyPixels)
```

:   Sets the projection matrix by using the glFrustrum() method of OpenGL.

---


### aim

```
public void aim(GL gl)
```

:   Uses gluLookAt() to position and aim the camera.

---


### resetDistance

```
public void resetDistance()
```

:   Sets the camera position based on the initial camera distance calculated
    when a new Structure is loaded. The Model is always placed centered on the
    origin, and the camera is placed looking down the z-axis at the origin.

---


### setInitialCameraDistance

```
public void setInitialCameraDistance(int z)
```

:   Sets the initial camera distance on the z-axis.

---


### reshape

```
public void reshape(GL gl,
                    int x,
                    int y,
                    int width,
                    int height)
```

:   Called on when the GLAutoDrawable object has changed its size so
    that the perspective and viewport can be recalculated.

    :   **Parameters:**: `gl` - the current GL object.: `x` - the x-coordinate of the canvas.: `y` - the y-coordinate of the canvas.: `width` - the width of the canvas.: `height` - the height of the canvas.

---


### moveX

```
public void moveX(int x)
```

:   Add to the camera x position.

    :   **Parameters:**: `x` - value to add to camera x-coordinate.

---


### moveY

```
public void moveY(int y)
```

:   Add to the camera y position.

    :   **Parameters:**: `y` - value to add to camera y-coordinate.

---


### moveZ

```
public void moveZ(int z)
```

:   Add to the camera z position.

    :   **Parameters:**: `z` - value to add to camera z-coordinate.

---


### printXYZ

```
public void printXYZ()
```

:   Prints the xyz-coordinates for the camera.

---


### printModelViewMatrix

```
public void printModelViewMatrix()
```

:   This debugging method prints the copy of the ModelViewMatrix
    stored n m\_modelViewMatrix.


---


|  |  |  |
| --- | --- | --- |
| **Overview** \| **Package** \| **Class** \| **Use** \| **Tree** \| **Deprecated** \| **Index** \| **Help** | |  |
| PREV CLASS   **NEXT CLASS** | **FRAMES**    **NO FRAMES**     **All Classes** |  |
| SUMMARY: NESTED \| FIELD \| CONSTR \| METHOD | DETAIL: FIELD \| CONSTR \| METHOD |  |


---

# *Copyright © 2007-2008*
